# Seroepidemiology of *Toxoplasma gondii *infection in psychiatric inpatients in a northern Mexican city

**DOI:** 10.1186/1471-2334-6-178

**Published:** 2006-12-19

**Authors:** Cosme Alvarado-Esquivel, Olga-Patricia Alanis-Quiñones, Miguel-Ángel Arreola-Valenzuela, Alfredo Rodríguez-Briones, Luis-Jorge Piedra-Nevarez, Ehecatl Duran-Morales, Sergio Estrada-Martínez, Sergio-Arturo Martínez-García, Oliver Liesenfeld

**Affiliations:** 1 Faculty of Medicine, Juárez University of Durango State (UJED). Durango, Mexico; 2 Hospital of Mental Health "Dr. Miguel Vallebueno", Durango City, Secretary of Health. Durango, Mexico; 3 State Center for Blood Transfusion. Durango City, Secretary of Health. Durango, Mexico; 4 Institute for Scientific Research, UJED. Durango, Mexico; 5 Institute for Microbiology and Hygiene, Campus Benjamin Franklin, Charité Medical School Berlin, Germany

## Abstract

**Background:**

Patients with psychiatric disorders were found to show a high seroprevalence of *Toxoplasma gondii *infection. There is scarce information about the epidemiology of *T. gondii *infection in psychiatric patients in Mexico. Therefore, we sought to determine the prevalence of *T. gondii *infection and associated socio-demographic, clinical and behavioural characteristics in a population of psychiatric patients in Durango City, Mexico. Seroprevalence in patients was compared with that obtained in a control population.

**Methods:**

One hundred and thirty seven inpatients of a public psychiatric hospital and 180 controls were examined for the presence of IgG and IgM antibodies against *T. gondii *by enzyme-linked immunoassay (Diagnostic Automation Inc., Calabasas, CA, USA). The control population consisted of blood donors of a public blood bank and elderly persons attending a senior center in the same city. Age in controls (42 years +/- 20.2) was comparable with that of the psychiatric patients (43.7 years +/-13.8) (p = 0.42). Socio-demographic, clinical and behavioral characteristics from the patients were also obtained.

**Results:**

Anti-*T. gondii *IgG antibodies indicating latent infection with *T. gondii *was found in 25 (18.2%) of 137 psychiatric inpatients and 16 (8.9%) of 180 controls (p = 0.02). Ten (26.3%) of 38 schizophrenic patients had latent infection and this prevalence was also significantly higher than that observed in controls (p = 0.005). Prevalence of anti-*T. gondii *IgM antibodies was comparable among patients and controls (4.4% vs 2.2%, respectively, p = 0.22). Multivariate analysis showed that *T. gondii *infection in inpatients was positively associated with sexual promiscuity (adjusted OR = 15.8; 95% CI: 3.8–64.8), unwashed raw fruit consumption (adjusted OR = 5.19; 95% CI: 2.3–11.3), and a history of surgery (adjusted OR = 6.5; 95% CI: 2.6–16), and negatively associated with lamb meat consumption (adjusted OR = 0.26; 95% CI: 0.10–0.63).

**Conclusion:**

In the present study, psychiatric inpatients in Durango, Mexico, in general and schizophrenia inpatients in particular had a significantly higher prevalence of *T. gondii *infection than the control group. Results suggest that unwashed raw fruit consumption might be the most important route of *T. gondii *transmission in our psychiatric inpatients while lamb meat consumption the less important. Additional studies will have to elucidate the causative relation between infection with *T. gondii *and psychiatric disorders.

## Background

*Toxoplasma gondii *(*T. gondii*) is a coccidian parasite found worldwide [1,2], that infects nearly one third of humanity [2,3]. Humans acquire a *T. gondii *infection mainly by ingesting food or water that is contaminated with oocysts shed by cats or by eating undercooked or raw meat containing tissue cysts [3-5]. Primary infection acquired during pregnancy may result in severe damage to the foetus [3,6], and may cause mental retardation, seizures, blindness, and death [7]. For its part, acquired infections in humans are usually asymptomatic, but in some infected persons cervical lymphadenopathy or ocular disease may occur [1,3]. Acquired *T. gondii *infection in immunocompetent patients may also cause central nervous system manifestations as Guillain-Barré syndrome [8], or cause a brain abscess [9]. In addition, acquired acute toxoplasmosis may be associated with psychiatric manifestations [10]. Yolken *et al *[11] have shown that individuals with first-episode schizophrenia had significantly increased levels of antibodies against *T. gondii *as compared with control subjects. In a meta-analysis of 19 studies published since 1953 of *T. gondii *antibodies in persons with schizophrenia and other severe psychiatric disorders and in controls, researchers found that 11 studies reported a significantly higher percentage of antibodies in the affected persons [12]. Most of the reported seroprevalence studies in psychiatric patients were performed before 1980, and diagnostic methods used in these studies as skin test, complement fixation, and colour change in fish, are less specific than enzyme immunoassays used in the more recent epidemiological studies [12]. Similarly, risk factors for *T. gondii *infection in psychiatric patients have been poorly explored. Due to the limited number of recent epidemiological studies in psychiatric patients in general, and a lack of them in Mexico in particular, we have performed a cross-sectional study in order to determine the prevalence of *T. gondii *infection in psychiatric patients of Durango City, Mexico. We also included in our study investigations about the association between infection and the patient characteristics including sociodemographic and clinical data as well as known risk factors for infection. Results obtained were compared to a control population of blood donors and elderly persons attending a senior center in the same Durango City.

## Methods

### Study populations

We have studied two populations: psychiatric inpatients and control subjects.

#### Psychiatric inpatients

From December 2005 to March 2006, all inpatients of the public psychiatric hospital of Durango City, Mexico were invited to participate in the study. During the study period, 158 patients were hospitalized. Out of the 158 inpatients, 137 were included in the study based on the following criteria for inclusion: 1) psychiatric inpatients; 2) aged 16 years and older; and 3) who accepted to participate in the study. Twenty one patients were excluded because either they did not accept to participate, or did not provide blood for analysis or did not submit the questionnaire. Thirty nine were females and 98 were males. Inpatients had been hospitalised from 3 days to 46 years (mean: 9.1 years). Ninety three patients suffered from an acute psychiatric disease, and 44 from a chronic psychiatric disease. Recent admissions (length of stay up to 2 months) were documented in 39 inpatients.

#### Control subjects

Since psychiatric patients had a wide range of age, we included two control populations. One represented by 129 blood donors of a public blood bank and the other consisted of 51 elderly persons attending a senior center in Durango City. Inclusion criteria for blood donors were: 1) blood donors; 2) aged 16–54 years; and 3) who agreed to participate in the study. In this study, we considered a senior center as a place where the elderly attend courses such as handicraft, dancing, primary school, high school, singing, etc., and organize local and national travels for pleasure. In elderly persons, inclusion criteria were: 1) senior center attendees; 2) aged 55 years and older; and 3) who agreed to participate in the study. All controls were enrolled consecutively. Fifty five were females and 125 males. The mean age of the control subjects (42 years +/- 20.2) was comparable with that of the psychiatric patients (43.7 years +/-13.8) (p = 0.42).

### Socio-demographic, clinical and behavioural data

Socio-demographic data including age, birthplace, residence, marital status, occupation, educational level, socio-economic level and housing conditions index were obtained from all patients. Housing conditions index was obtained by using the Bronfman's criteria [13]. Briefly, five variables were evaluated: number of persons in the house, number of rooms in the house, material of the floor of the house, availability of drinkable water, and form of elimination of excretes. Clinical data including blood transfusion or transplant history; and behavioral data including animal contacts, cleaning up cat faeces, foreign travel, kind of meat consumption (pork, lamb, beef, goat, boar, chicken, turkey, rabbit, deer, squirrel, horse, sea food, snake and bird), raw or undercooked meat consumption, unpasteurized milk or milk products consumption, untreated water consumption, eating dried or cured meat (chorizo, ham, sausages or salami), unwashed raw vegetables or fruits consumption, contact with soil (gardening or agriculture), and eating outside of the home from the participants were obtained. Data was obtained from the patients, medical examination records, and informants. Patients were invited to provide veridical information and they were informed that data were used in a confidential manner. Classification of mental illnesses was performed according to the ICD-10 criteria [14].

### Laboratory test

Sera of the participants were analysed for anti-*T. gondii *IgG antibodies by a commercially available enzyme immunoassay "Toxoplasma IgG" kit (Diagnostic Automation Inc., Calabasas, CA, USA). In addition, sera positive for anti-*T. gondii *IgG antibodies were further analysed for anti-*T. gondii *IgM antibodies by a commercially available enzyme immunoassay "Toxoplasma IgM" kit (Diagnostic Automation Inc., Calabasas, CA, USA).

### Ethical aspects

This study was approved by the ethical committee of the Hospital of Mental Health "Dr. Miguel Vallebueno", in Durango City. The purpose and procedures of the study were explained to all participants, and a written informed consent was obtained from all of them.

### Statistical Analysis

Results were analyzed with the aid of the software Epi Info 6 and SPSS 7.0. Age among the groups was compared by the student's *t *test. For comparison of the frequencies among the groups, the Yates corrected test and when indicated the Fisher exact test, were used. Bivariate and multivariate analyses were used to assess the association between the characteristics of the subjects and *T. gondii *infection. Variables were included in the multivariate analysis if they had a p value of less than 0.20 in the bivariate analysis. Adjusted odd ratio (OR) and 95% confidence interval (CI) were calculated by multivariate analysis using logistic regression.

## Results

### Seroprevalence of anti-*T. gondii *antibodies

Twenty-five (18.2%) of 137 psychiatric inpatients and 16 (8.9%) out of the 180 controls were positive for anti-*T. gondii *IgG antibodies indicating latent infection with *T. gondii*. Table 1 shows the seroprevalences of latent *T. gondii *infection in the populations studied according to age groups. Seroprevalence in patients and controls increased with age (Table 1). However, patients in most age groups showed higher rates of seroprevalence; whereas the differences in individual age groups did not reach statistical significance, the difference among these prevalences in all age groups taken together was statistically significant (p = 0.02).

**Table 1:** Seroprevalence of anti-*T. gondii *IgG antibodies in psychiatric patients and controls according to age groups.

	Psychiatric patients			Controls			
		*T. gondii *infection			*T. gondii *infection		
Age groups	No. tested	No.	%	No. tested	No.	%	P value
16–24	8	0	0	40	2	5	0.69
25–34	30	5	16.7	48	3	6.3	0.13
35–44	27	4	14.8	32	4	12.5	0.54
45–54	33	8	24.2	9	1	11.1	0.36
55–64	17	5	29.4	8	1	12.5	0.34
65–74	6	0	0	31	5	16.1	0.38
75–84	4	1	25	12	0	0	0.25
							
16–84	125	23	18.4	180	16	8.9	0.02
							
Unknown age	12	2	16.7				
							
All	137	25	18.2	180	16	8.9	0.02

Seroprevalence in patients with a long history of mental disease was 25% (11/44) while seroprevalence in those with recent onset of their mental illness was 15.1% (14/93) (p = 0.24). Latent *T. gondii *infection was significantly higher in schizophrenics (10/38, 26.3%) than in controls (16/180, 8.9%) (p = 0.005). Table 2 shows the seroprevalences of *T. gondii *infection in the inpatients according to their psychiatric disorder. The highest prevalence of latent *T. gondii *infection in schizophrenics was found in patients aged 45–54 years old (7/17, 41.2%) (Table 3). Latent *T. gondii *infection was found in 3 (20%) of 15 schizophrenics females and 7 (30.4%) of 23 schizophrenic males. Anti-*T. gondii *antibodies were found in 5 (25%) of 20 acute schizophrenic patients and in 5 (27.8%) of 18 chronic schizophrenic patients. With respect to anti-*T. gondii *IgM antibodies in persons with anti-*T. gondii *IgG antibodies, we observed seropositivity in 6 (4.4%) psychiatric patients, 4 (2.2%) blood donors, and none of the senior center attendees. No statistically significant difference in seroprevalence of anti-*T. gondii *IgM antibodies was found between psychiatric patients and controls.

**Table 2:** Clinical diagnosis and seroprevalence of anti-*T. gondii *IgG antibodies in 137 psychiatric patients.

Clinical Diagnosis	ICD-10 diagnosis	Patients studied		Patients with anti-*T. gondii *antibodies		
		No.	%	No.	%	P value*
Schizophrenia	F20	38	27.7	10	26.3	0.005
Mental and behavioural disorders due to psychoactive substance use	F14, F18–19	25	18.2	4	16	0.21
Mental disorder	F06.2, F06.8–9	20	14.6	1	5	0.47
Mental retardation	F71–73	19	13.9	4	21.1	0.1
Mental and behavioural disorders due to use of alcohol	F10	14	10.2	2	14.3	0.38
Bipolar affective disorder	F31	6	4.4	1	16.7	0.44
Epilepsy	G40	4	3	0	0	0.69
Severe depressive episode	F32.2–3	3	2.2	1	33.3	0.25
Dementia in Alzheimer's disease with early onset	F00.0	2	1.5	2	100	0.009
Dissociative disorder	F44	1	0.7	0	0	0.91
Vascular dementia	F01	1	0.7	0	0	0.91
Somatoform disorder	F45	1	0.7	0	0	0.91
Obsessive-compulsive disorder	F42	1	0.7	0	0	0.91
Acute polymorphic psychotic disorder	F23.1	1	0.7	0	0	0.91
Schizoaffective disorder, mixed type	F25.2	1	0.7	0	0	0.91
						
All		137	100	25	18.2	0.02

*As compared with 8.9% seroprevalence of anti-*T. gondii *IgG antibodies in controls (16/180).

**Table 3:** Seroprevalence of anti-*T. gondii *IgG antibodies in schizophrenic patients and controls according to age groups.

	Schizophrenic patients			Controls			
		*T. gondii *infection			*T. gondii *infection		
Age groups	No. Tested	No.	%	No. tested	No.	%	P value
25–34	5	0	0	48	3	6.3	0.73
35–44	6	1	16.7	32	4	12.5	0.59
45–54	17	7	41.2	9	1	11.1	0.12
55–64	5	1	20	8	1	12.5	0.64
65–74	2	0	0	31	5	16.1	0.71
75–84	3	1	33.3	12	0	0	0.2
							
All	38	10	26.3	140	14	10	0.01

### Factors associated with seropositivity

In the bivariate analysis, seven variables were identified as possible risk factors associated with *T. gondii *infection: 1) sexual promiscuity (0.04); 2) unwashed raw fruit consumption (p = 0.06); 3) deer meat consumption (p = 0.07); 4) a history of surgery (p = 0.08), 5) being born outside Durango State (p = 0.10); 6) untreated water consumption (p = 0.20); and 7) eating outside of the home (p = 0.20). In addition, we found two variables that showed possible negative association with *T. gondii*: 1) lamb meat consumption (p = 0.06); and 2) pork meat consumption (p = 0.18). The rest of the sociodemographic, clinical and behavioural characteristics of patients did not show any association with *T. gondii *infection. Multivariate analysis (Table 4) revealed that only three variables were associated with *T. gondii *seropositivity: 1) sexual promiscuity (adjusted OR = 15.8; 95% CI: 3.8–64.8); 2) unwashed raw fruit consumption (adjusted OR = 5.19; 95% CI: 2.3–11.3); and 3) a history of surgery (adjusted OR = 6.5; 95% CI: 2.6–16). In addition, the multivariate analysis revealed that only lamb meat consumption was negatively associated with *T. gondii *infection (adjusted OR = 0.26; 95% CI: 0.10–0.63).

**Table 4:** Multivariate analysis of characteristics of the psychiatric patients and their association with *T. gondii *infection.

Characteristic^a^	Adjusted odds ratio^b^	95% Confidence interval	P value
Sexual promiscuity	15.8	3.8–64.8	0.0001
Unwashed raw fruit consumption	5.19	2.3–11.3	0.01
Deer meat consumption	0.93	0.37–2.30	0.87
Surgery history	6.5	2.6–16	<0.000
Being born outside Durango State	1.25	0.66–2.36	0.48
Untreated water consumption	1.4	0.72–2.86	0.29
Eating outside of the home	0.93	0.61–1.42	0.76
Lamb meat consumption	0.26	0.10–0.63	0.003
Pork meat consumption	0.37	0.11–1.17	0.09

^a^The variables included were those with a p < 0.20 obtained in the bivariate analysis.

^b^Adjusted by gender and age.

## Discussion

In this study, we found an 18.2% prevalence of *T. gondii *infection in psychiatric inpatients of Durango City, Mexico. Reports on the prevalence of *T. gondii *infection among populations of psychiatric inpatients are scarce. In a previous Mexican study, Buentello [15] reported a significantly higher prevalence of *T. gondii *infection in inpatients with schizophrenia (29/42, 69%) than in normal subjects (0/60, 0%). In a Cuban study, a 60% prevalence of *T. gondii *infection was reported in psychiatric patients admitted to wards of a teaching hospital and this prevalence was significantly higher than the one observed in healthy subjects [16]. The prevalence found in our study is much lower than that reported in the Cuban study. Nevertheless, in both studies it became clear that psychiatric patients showed significantly higher prevalences of *T. gondii *infection than the control groups. The difference in the prevalences among these studies might be explained by a difference in the prevalence found in the general population in each studied city. We recently found a prevalence of 6.1% in pregnant women in Durango City [1] and 8.9% in our control population of the present study whereas in the Cuban study the control healthy subjects had a prevalence of 30% [16]. The difference in the prevalence among the psychiatric and control populations might be explained, at least in part, by differences in the sanitary conditions among the groups. Indeed, most psychiatric inpatients belonged to a lower socio-economic level and had lower housing conditions than the control populations. Nevertheless, comparison of socio-economic status among infected and not infected patients did not show any statistically significant difference. We did not find any difference in seroprevalence between patients with a long history of mental disease versus those with recent onset of their mental illness. Although the prevalence of anti-*T. gondii *IgM antibodies was double in patients than controls, no statistically significant difference was reached. This was most likely due to the low numbers of positive subjects among the groups. When specific diagnosis of psychiatric diseases were analysed individually with respect to the prevalence of *T. gondii *infection, we found a significantly higher prevalence in schizophrenic patients than in the control population (p = 0.005). However, we did not find any statistically significant difference in prevalence when age groups were compared. This might be explained by the limited size of the sample in each age group. The overall significantly higher seroprevalence found in our schizophrenia patients than our controls supports an association between *T. gondii *infection and schizophrenia as other researchers have reported. Yolken *et al *[11] showed that individuals with first-episode schizophrenia had significantly increased levels of antibodies against *T. gondii *as compared with control subjects. Delgado-Garcia *et al *[17,18] found that the highest percentage of skin-test-positive patients were the most advanced schizophrenics, and the more severe the patient state the higher the intensity of the test reactivity. Wang *et al *[19] showed that the seroprevalence of anti-*T. gondii *IgG but not IgM-antibodies in schizophrenia patients was higher than the seroprevalence in control groups. Similarly, in a large study, Roch and Varela [20] showed that schizophrenia patients had a significantly higher prevalence of *T. gondii *infection than subjects of the general population (836/973, 86% vs 4411/14689, 30%, respectively). Interestingly, all Alzheimer patients included in the study had IgG-antibodies against *T. gondii*. Since the number of patients was rather small, further studies are needed to clarify this observation.

When the socio-demographic and behavioral characteristics of the psychiatric patients were analysed by logistic regression, we found that *T. gondii *infection was associated with sexual promiscuity (adjusted OR = 15.8; 95% CI: 3.8–64.8). Interpretation of this finding should be taken with care since the CI of the OR was wide. In addition, this finding is surprising since the sexual route seems not to be effective in parasite transmission in men and animals [21]. Nevertheless, this route can not be absolutely excluded since *T. gondii *has been found in male genital tract [22]. In addition, a history of surgery (adjusted OR = 6.5; 95% CI: 2.6–16) was associated with infection. This finding is also unexpected since none of the patients had undergone transplantation and parasite transmission by surgery other than transplantation has not been described. Most likely, confounders have contributed to the association of *T. gondii *infection with sexual promiscuity and a history of surgery. Nevertheless, it raises the question whether these characteristics might reflect parasite-induced behavioral and clinical changes in infected individuals. *T. gondii*-induced behavioral changes have been reported in rats. Berdoy *et al *[23] showed in wild/laboratory hybrid rats that the propensity to explore novel stimuli in their environment was higher in *T. gondii *infected than uninfected rats. In addition, Webster *et al *[24] showed that in wild rats with naturally occurring *T. gondii *infection low neophobia was significantly associated with positive *Toxoplasma *titers. Also in our study, we found that unwashed raw fruit consumption was associated with infection (adjusted OR = 5.19; 95% CI: 2.3–11.3), and this finding suggests that contaminated fruit probably by cat feces might also be important in parasite transmission in psychiatric patients. Known factors associated with *T. gondii *infection in other populations as contact with cats [25], drinking untreated water [26-28], contact with garden soil [29], and consumption of cured meat [30], were not associated with *T. gondii *infection in our study. On the other hand, we found that *T. gondii *infection in the psychiatric patients was negatively associated with lamb meat consumption (adjusted OR = 0.26; 95% CI: 0.10–0.63). This finding suggests that consumption of this meat played no role in parasite transmission in our psychiatric patients.

## Conclusion

We conclude that psychiatric inpatients in general and schizophrenia inpatients in particular had a significantly higher prevalence of *T. gondii *infection than the control group. Results suggest that unwashed raw fruit consumption indicating environmental contamination with oocysts might be the most important route of *T. gondii *transmission in our psychiatric inpatients. Additional studies will have to elucidate the causative relation between infection with *T. gondii *and psychiatric disorders.

## Competing interests

The author(s) declare that they have no competing interests.

## Authors' contributions

CAE conceived and designed the study protocol, participated in the coordination and management of the study, performed the analysis of the serum samples and data analysis, and wrote the manuscript. OPAQ designed the study protocol, and applied the questionnaires. MAAV applied the questionnaires and performed clinical evaluations. ARB applied the questionnaires in blood donors and performed the analysis of the serum samples. LJNP applied the questionnaires in blood donors. EDM applied the questionnaires in blood donors. SEM performed the statistical analysis. SAMG performed the analysis of the serum samples and monitored the study. OL designed the study protocol, performed the data analysis, and wrote the manuscript. All authors read and approved the final manuscript.

## Pre-publication history

The pre-publication history for this paper can be accessed here:


